# Global disease burden of uncorrected refractive error among adolescents from 1990 to 2019

**DOI:** 10.1186/s12889-021-12055-2

**Published:** 2021-11-01

**Authors:** Zhenlan Yang, Guangming Jin, Zijing Li, Yunru Liao, Xiang Gao, Yichi Zhang, Yuqing Lan

**Affiliations:** 1 Department of ophthalmology, Guangdong Provincial Key Laboratory of Malignant Tumor Epigenetics and Gene Regulation, Sun Yat-sen Memorial Hospital, Sun Yat-sen University, 107 Yanjiang West Road, Guangzhou, 510000 People’s Republic of China; 2 State Key Laboratory of Ophthalmology, Zhongshan Ophthalmic Center, Sun Yat-sen University, Guangzhou, China

**Keywords:** Uncorrected refractive error, Adolescents, Disability-adjusted life years, Socioeconomic, Urbanization, Education

## Abstract

**Background:**

To estimate the global disease burden of uncorrected refractive error (URE) among adolescents and assess the contributions of various risk factors to disability-adjusted life-years (DALYs) due to URE.

**Methods:**

Global, regional and country-level DALY numbers and rates due to URE among adolescents were acquired from the Global Burden of Disease Study 2019 database. Human Development Index (HDI), Socio-Demographic Index (SDI) and other country-level data were obtained from other open databases as potential indicators. Regression analysis was used to evaluate associations between DALY rates among adolescents and potential predictors.

**Results:**

Global DALYs due to URE among adolescents rose by 8% between 1990 and 2019 but moderately decreased by 4.8% during this period after adjusting for population size. Female adolescents showed higher DALY rates. DALY rates sharply increased from 5 to 9 years of age, then rose more slowly, reaching a plateau before 20 years of age. Country-level DALY rates in 2019 were positively associated with HDI, SDI, and urbanization rates but negatively correlated with primary school dropout rates. Higher disease burden of adolescents visually impaired from URE was associated with lower primary school dropout rates (β = − 0.257, 95% CI − 0.376 to − 0.138, *P* < 0.001) and higher urbanization rates (β = 0.257, 95% CI 0.067 to 0.256, *P* = 0.001).

**Conclusions:**

Higher socioeconomic status, urbanization rates and education levels are associated with a heavier disease burden of URE among adolescents. The findings of this study can provide a reference for policy making on resource allocation for URE prevention and control in teenagers.

## Background

Uncorrected refractive error (URE) is an underappreciated but widespread public health problem that significantly diminishes both quality of life and productivity. Among the global population with vision impairment and blindness in 2015, URE resulted in moderate to severe visual impairment in 116.3 million people and blindness in 7.4 million people. With an extremely high prevalence, URE ranks first among visual impairment causes, and the affected population is expected to continually grow [1]. Being at the critical period of eye development,children and adolescents could be sensitive to environmental factors and at high risk of suffering from refractive disorders. Especially during the coronavirus disease 2019 (COVID-19) pandemic period, these school-aged children could be vulnerable to undiagnosed myopia owing to the home confinement policy and not attending class where myopic individuals can be highlighted through routine lessons (e.g. copying from the board) [2]. Access to ophthalmology outpatient services including optometry were also limited during the peaks of the pandemic, and the need for these services is now backlogged.

Given the difficulties that people with URE face in activities of daily living and social functioning, it is not surprising that URE can be associated with lower quality of life [3] and productivity loss [4]. The adverse effects of URE can be more serious in children, whose poor-quality visual experience due to URE can lead to unreversible amblyopia [5] and inadequate knowledge acquisition from the outer world during development and maturation [6]. There is no doubt that good visual performance has a profound effect on children’s cognitive and psychosocial development [7, 8].

Although there have been many studies on the epidemiology and etiology of refractive error, there are few evaluations quantifying its health burden. The Global Burden of Diseases Study (GBD study) [9] developed disability-adjusted life years (DALYs) to estimate the disease burden of many conditions, including visual impairment due to URE. DALYs are determined by the number of disabled years weighted by the level of disability caused by a disability or disease. Previous research has examined the DALYs of URE among people of all ages [10]. However, an aged population was generally affected by presbyopia and the previous study failed to fully describe the URE burden among adolescents. As most URE in adolescents is preventable and controllable, in this study, we focused on the disease burden of URE in adolescents [11–13]. The aim of this study was to describe the temporal trends of the global disease burden of URE among adolescents and its distribution across different age brackets, sexes, and regions and to explore the risk factors for DALYs due to URE in adolescents, including national-level data on demographic, socioeconomic and educational factors.

This research has been conducted as part of the Global Burden of Diseases, Injuries, and Risk Factors Study (GBD), coordinated by the Institute for Health Metrics and Evaluation. The GBD was partially funded by the Bill & Melinda Gates Foundation; the funders had no role in the study design, data analysis, data interpretation, or writing of the report.

## Methods

### Global disease burden of URE among adolescents

DALY data of URE were retrieved from the open database of the Global Burden of Disease 2019 Study in the Global Health Data Exchange (GHDx; http://ghdx.healthdata.org/gbd-results-tool), which aggregated DALYs due to 369 diseases and injuries for 204 countries and territories from 1990 to 2019. International Classification of Diseases (10th edition) codes H52.0–H52.7 were mapped to URE covering myopia, hypermetropia, astigmatism, anisometropia and aniseikonia, presbyopia, disorders of accommodation, other disorders of refraction and unspecified disorder of refraction. in the database. The methodology has been concretely illustrated in a previous study [9]. Overall, DALYs are calculated as the sum of years lived with disability (YLD) and years of life lost (YLL). As a nonfatal disease, URE’s DALY number = YLD = the number of prevalent cases × the disability weight. Disability weights, which represent the magnitude of health loss associated with specific health outcomes, are measured by standardized surveys containing lay descriptions of the major functional consequences and symptoms associated with diverse health states. As for URE, the disability weights vary with different degrees of vision impairment due to URE (specific values are available in the GHDx repository, http://ghdx.healthdata.org/record/ihme-data/gbd-2019-disability-weights). Based on GBD standard population structure, the DALY rate refers to the number of cases per 100,000 population (controlling for population size).

The following data were extracted from the database for further analysis: (1) the global DALY numbers, rates and global prevalence numbers and rates among adolescents (0–20 years old) in 1990–2019, (2) global sex-specific DALY numbers and rates from 1990 to 2019 and their age distribution (four age groups: 1 to 4, 5 to 9, 10 to 14 and 15 to 19 years of age) in 2019; (3) global maps of DALY numbers and rates among adolescents in 2019, with the top ten countries ranked by DALY numbers and rates. Global maps were generated from a data visualization tool available from the GHDx, supported by the Institute for Health Metrics and Evaluation (IHME) (https://vizhub.healthdata.org/gbd-compare/); and (4) World Health Organization (WHO) income level, the World Bank income level and Socio-Demographic Index (SDI)-level regional DALY rates in 2019.

### Country-level indicators

We assessed the relationship of country-specific DALY rates with several country-level demographic, socioeconomic and educational indicators derived from the following well-known open databases. The Human Development Index (HDI), primary school dropout rates and urbanization rates were obtained from the open database of the United Nations Development Programme (http://hdr.undp.org/en/data). 2019 SDI data were obtained from the GHDx open database (http://ghdx.healthdata.og/). HDI and SDI are two composite indicators of a country’s socioeconomic condition. The HDI is the geometric mean of the three-dimensional indices: health index, education index and income index. In details, the health index is calculated by life expectancy at birth, the income index is calculated by gross national income (GNI) per capita and the education index takes both average and expected years of schooling into account. Countries were classified into four groups according to HDI: low (0–0.550), medium (0.550–0.699), high (0.700–0.799), and very high (0.800–1) human development. The SDI is comparable to the HDI, but taking health index out of the computational equation. In short, it is the geometric mean of 0 of total fertility rate for those younger than 25 years old (TFU25), mean education for those 15 years old (EDU15+) and lag-distributed income (LDI) per capita. Moreover, the primary school dropout rate is defined as the percentage of students from a given cohort who have enrolled in primary school but who drop out before reaching the last grade of primary education. The urbanization rate is calculated as the proportion of population living in the areas classified as urban according to the criteria used by each country or area.

### Statistical analysis

The outcomes included the time, age, sex, and geographic distribution of DALYs due to URE. The Wilcoxon signed rank test was used to compare sex differences in DALY numbers and rates. One-way analysis of variance (ANOVA) was used to compare DALY rates in different WHO regions. The Kruskal-Wallis H test was used to investigate differences in DALY rates across four income-based and five SDI-based country groups, followed by the Mann-Whitney U test for post hoc pairwise comparisons. Scatter plots, Pearson correlation and univariate linear regression analyses were performed to explore the relationships between diverse demographic, socioeconomic and educational variables and DALY rates of uncorrected refractive disorder at the country level. Indicators that were significant in univariate analyses were further applied to stepwise multiple linear regression analyses. Statistical analyses were performed using SPSS software (SPSS, Inc.; Chicago, IL, USA; version 25.0), with *P* < 0.05 considered statistically significant. Figures were drawn using GraphPad Prism software (version 7.0e, GraphPad Software; San Diego, CA, USA).

## Results

### Trends in the disease burden of URE from 1990 to 2019

**Fig. 1:**
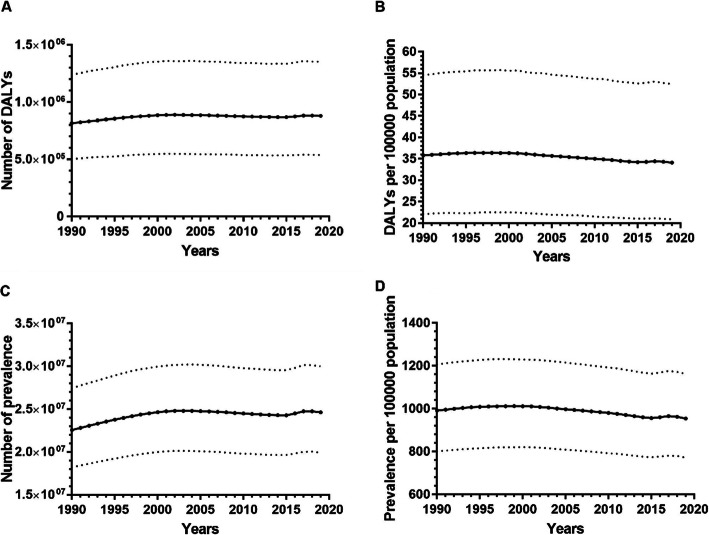
Time trends of global health burden and prevalence of URE among adolescents in 1990–2019. Panel A. DALY numbers. Panel B. DALY rates. Panel C. Prevalence numbers. Panel D. Prevalence rates. Dashed lines represent 95% CIs; DALY, disability-adjusted life year; URE, uncorrected refractive error

According to the GBD 2019, the number of DALYs due to URE slightly increased by 8%, from 814,261.98 (95% uncertainty interval [UI] 502,307.00–1,239,889.76) in 1990 to 879,736.05 (95% UI 538,302.00–1,353,108.75) in 2019 (Fig. 1A). Conversely, the DALY rate exhibited a slight downward trend, decreasing by 4.8%, from 35.81 (95% UI 22.09–54.53) to 34.11 (95% UI 20.87–52.46) (Fig. 1B). In addition, we observed that the numbers and rates of URE prevalence have remained high and relatively stable over the past 30 years (Fig. 1C and D).

### Global disease burden of URE by age and sex

**Fig. 2:**
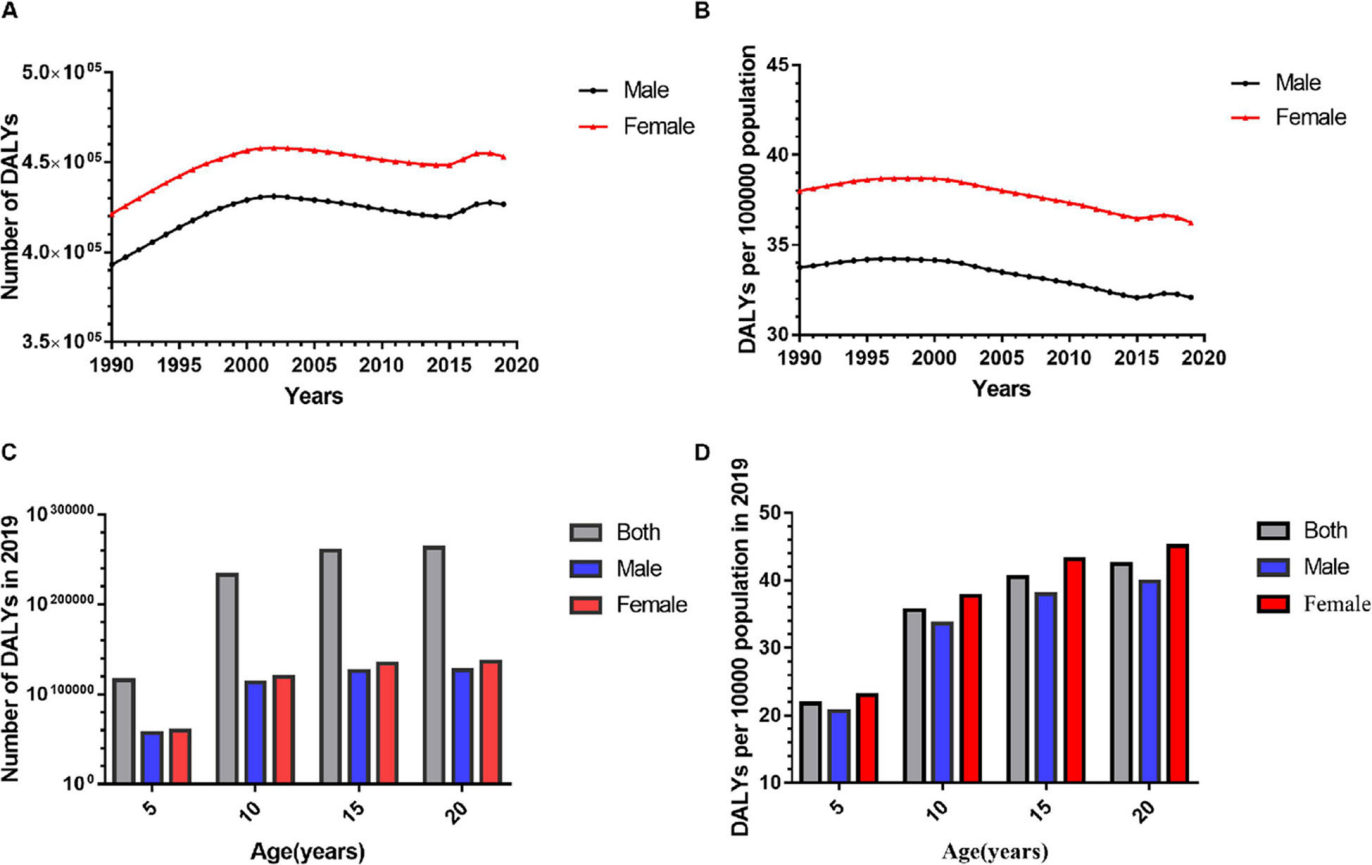
The distribution of global burden due to URE among adolescents by age and sex. Panel A. Sex-specific DALY numbers. Panel B. Sex-specific DALY rates. Panel C. Age-specific and sex-specific DALY numbers in 2019. Panel D. Age-specific and sex-specific DALY rates in 2019. DALY, disability-adjusted life year; URE, uncorrected refractive error

Wilcoxon signed-rank tests showed that there were significant sex disparities, as total DALYs for women significantly exceeded those of men from 1990 to 2019 (for both DALY numbers and rates, *P*<0.001) (Fig. 2 A and B). Figure 2 C and D showed the global DALY variation by sex in different age groups in 2019. The two sexes showed a similar trend in global DALY rates by age, sharply increasing from 5 to 9 years of age, then slowly rising, and reaching a plateau before 20 years of age. Wilcoxon signed rank tests showed that there were no significant sex disparities in the global DALY rates for each age group (*P* = 0.068).

### Regional differences in global disease burden due to URE

**Fig. 3:**
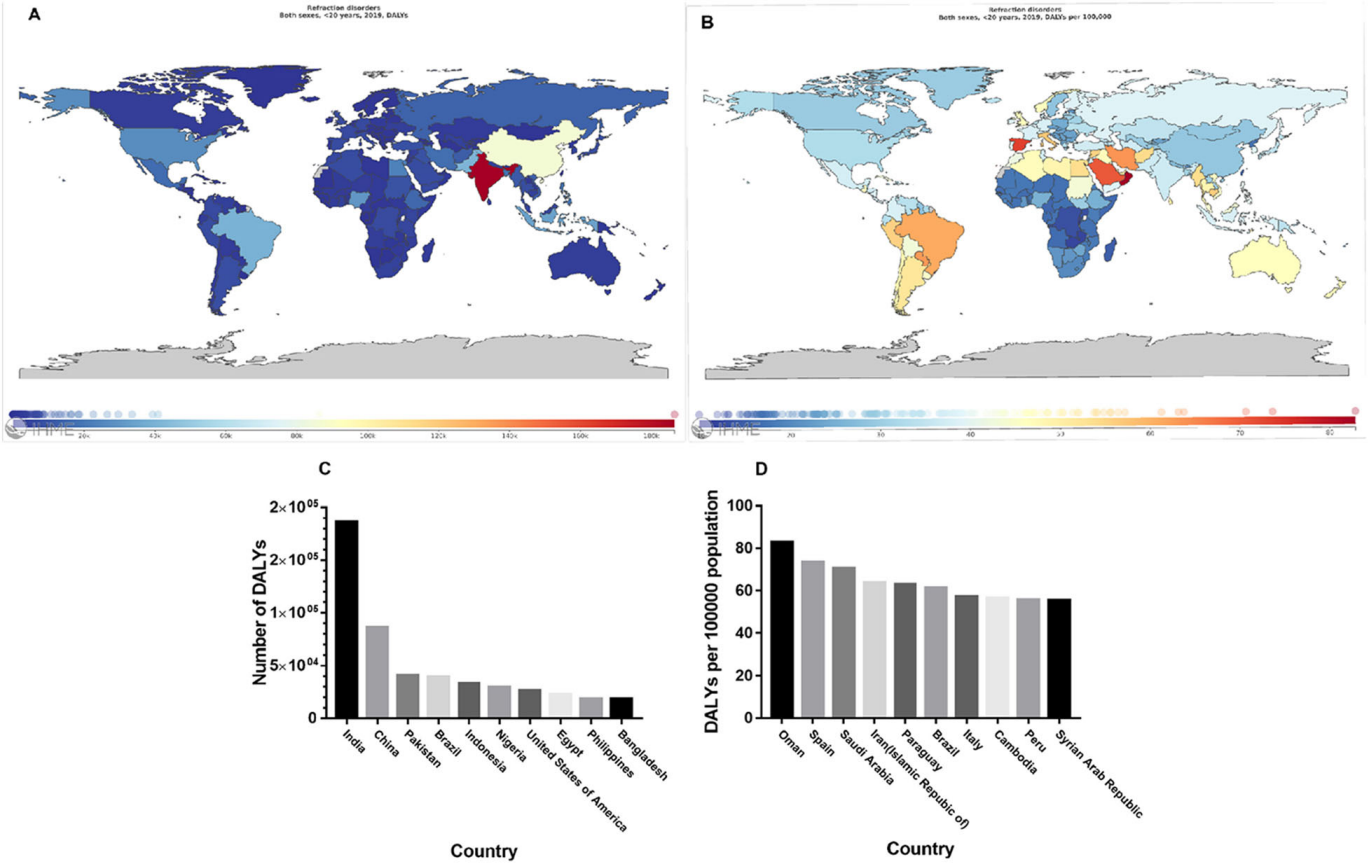
Global map of the disease burden of adolescents visually impaired from URE. Global maps were generated from a data visualization tool available from the Global Health Data Exchange (GHDx) repository (https://vizhub.healthdata.org/gbd-compare/). Panel A. DALY numbers. Panel B. DALY rates. Panel C. The 10 countries with the highest DALY numbers. Panel D. The 10 countries with the highest DALY rates. DALY, disability-adjusted life years; URE, uncorrected refractive error

**Fig. 4:**
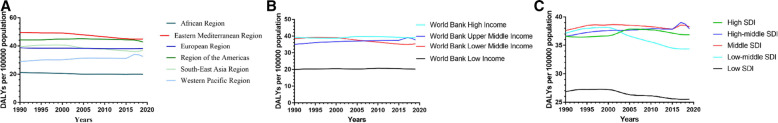
Global health burden distribution of URE among adolescents in different regions. Panel A. WHO regions. Panel B. Different SDI-level regions. All estimated GBD 2019 locations are divided into five groups referring to SDI quintile values: High SDI (0.805–1), High-middle SDI (0.690–0.805), Middle SDI (0.608–0.690), Low-middle SDI (0.455–0.608), Low SDI (0–0.455). Panel C. Different income-level regions. All estimated GBD 2019 locations are divided into four income groups according to GNI per capita, calculated using the World Bank Atlas method: High income ($12,696 or more), Upper middle income ($4096–$12,695), Lower middle income ($1046–$4095), Low income ($1045 or less). WHO, World Health Organization; SDI, Socio-Demographic Index; GNI, gross national income; URE, uncorrected refractive error

The severity of disease burden due to URE varies worldwide (Fig. 3 A and B). As expected, the countries with the largest populations had the highest DALY numbers (Fig. 3 C). India had the greatest total number of DALY due to URE (186,533.31, 95% UI 114,179.97–287,138.60). After adjusting for population size, the DALY rate was highest in Oman (82.85, 95% UI 49.99–128.59) (Fig. 3 D). One-way ANOVA and the Bonferroni correction for multiple comparisons indicated that the Eastern Mediterranean region had the heaviest disease burden compared to the remaining five WHO regions over the past 30 years (*P* < 0.001), followed by the Region of the Americas. The African Region had the lightest burden. All time trends for rates in the six WHO regions remained steady from 1990 to 2019(Fig. 4 A). Each of the income-level and SDI-level regions were also analyzed. The DALY rates were highest in high-income regions and lowest in low-income regions (Fig. 4 B). Middle SDI and low SDI regions ranked first and last, respectively, in terms of the DALY rates, while in recent years, high-middle SDI regions passed middle SDI regions and took first place (Fig. 4 C).

### Country-level disease burden due to URE and associations with national indicators

**Fig. 5:**
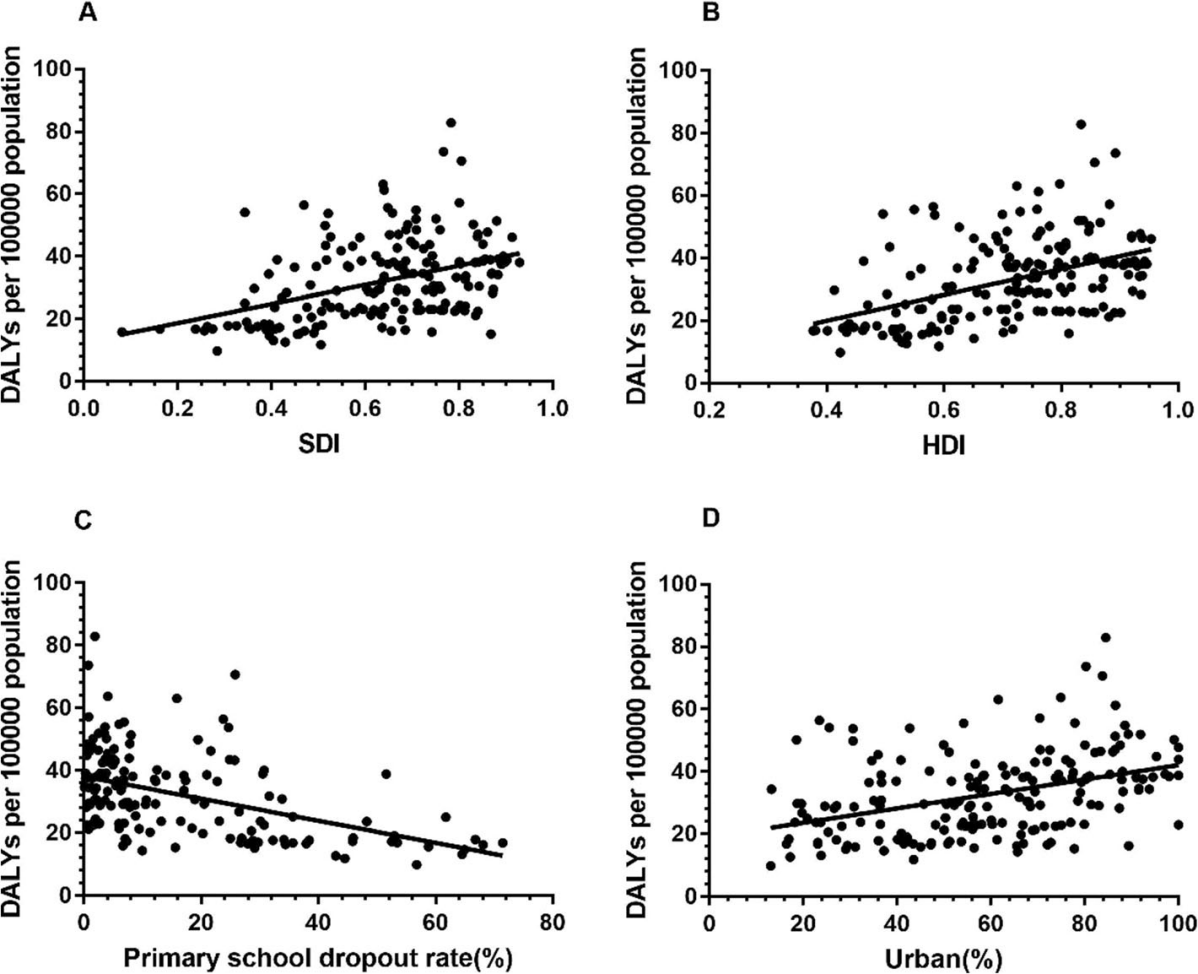
Relationship between the disease burden of URE among adolescents and country-level indicators. Panel A. SDI. The SDI is the geometric mean of total fertility rate for those younger than 25 years old (TFU25), mean education for those 15 years old (EDU15+) and lag-distributed income (LDI) per capita. Panel B. HDI. The HDI is the geometric mean of life expectancy at birth, gross national income (GNI) per capita and average and expected years of schooling. Panel C. Primary school dropout rates. It is calculated as the percentage of students from a given cohort who have enrolled in primary school but who drop out before reaching the last grade of primary education. Panel D. Urbanization rates. It is calculated as the proportion of population living in the areas classified as urban according to the criteria used by each country or area. HDI, Human Development Index; SDI, Socio-Demographic Index

The scatter plots between the disease burden of URE and country-level indictors are presented in Fig. 5. Linear trends were observed between the disease burden of URE and SDI, HDI, primary school dropout rate and urbanization rate. Pearson correlation and linear regression analysis revealed that the country-level SDI, HDI and urbanization rate were significantly positively correlated with the DALY rates of URE, while primary school dropout rates were negatively correlated with DALY rates (Table 1). Upon further analysis, primary school dropout rates and HDI were the most influential indicators. In stepwise multiple linear regression analyses, higher urbanization rates (β = 0.257, 95% CI 0.067–0.256, *P* = 0.001) and lower primary school dropout rates (β = − 0.257, 95% CI − 0.376 to − 0.138, *P* < 0.001) were associated with a higher disease burden resulting from URE (Table 1).

**Table 1:** Linear regression analysis of the relationship between country-level disease burden of URE and socioeconomic variables

Factors	Univariate linear regression			Multiple linear regression		
	Adjusted R square	β(95% CI)	*P* value	Adjusted R square	β(95% CI)	*P* value
SDI	0.167	30.548 (20.637 to 40.459)	<0.001*	–	–	–
HDI	0.206	41.17 (29.347 to 53.001)	<0.001*	–	–	–
Primary school dropout rate (%)	0.207	−0.355 (− 0.463 to − 0.247)	<0.001*	0.257	−0.257 (− 0.376 to − 0.138)	<0.001*
Urban (%)	0.155	0.231 (0.154 to 0.308)	<0.001*	0.257	0.162 (0.067 to 0.256)	0.001*

SDI – Socio-Demographic Index, HDI – Human Development Index

## Discussion

Refractive errors are the result of a mismatch between the axial length of the eye and its optical power, creating blurred vision. Eye growth, namely, emmetropization, is believed to be completed at the age of 13 years, while axial length growth in myopic eyes may continue [14]. Myopes typically exhibit early progression quickly, and the progression then slows down; in general, their refractive error eventually stabilizes during late adolescence, e.g., 15–21 years of age [15]. High incidence and high degrees of astigmatism were demonstrated to exist among children, especially newborns. As children grow older, the cornea flattens with significantly reduced astigmatism and stabilizes before adulthood [16]. Therefore, in this study, adolescence was defined as ages below 20 years.

This study used DALYs for the first time to elucidate the disease burden associated with URE among adolescents, exploring temporal trends from 1990 to 2019 and the global distribution by age, sex, WHO regions, levels of income and SDI. From 1990 to 2019, the world DALY numbers due to URE increased by 8%, while the world DALY rates slightly decreased over time after adjusting for increases in population. The global DALY rates of URE increased by age. Females had higher DALY rates than males of the same age. Regarding regional distribution, the disease burden in the Eastern Mediterranean, high-income and middle SDI regions was highest. Further research revealed that the disease burden of URE was associated with HDI, SDI, primary school dropout rates and urbanization rate. Stepwise linear multiple regression revealed that after eliminating collinearity, only primary school dropout rates and urbanization rates were significantly correlated with the disease burden of URE.

The results showed fluctuation in the prevalence and disease burden of URE among adolescents from 1990 to 2019. The temporal variation in the disease burden of URE was largely in accordance with the variations observed in prevalence. However, compared to prevalence, DALYs are more suitable indicators to measure the influence of a disease on quality of life. The total DALY numbers due to URE increased from 1990 to 2019, while the DALY rates remained stable after adjusting for global population growth. The high disease burden of URE among adolescents persists, suggesting that current health policies to control URE have failed to alleviate the additional disease burden caused by increasing prevalence. According to Holden et al. [17], the global myopic population will continue to expand and reach 4.758 billion (49.8% of the world population) by 2050, which means healthcare planners will face an even heavier burden of visual impairment related to URE.

Sex inequality in the global burden of URE exists even among adolescents and has persisted since 1990, with females bearing a significantly higher burden of URE than males, as has been reported previously [18]. Many studies have revealed a higher prevalence of refractive disorders among young females than in males. This may reflect different environmental risk factors, such as the tendency of girls to spend a greater number of hours engaged in near vision activities and significantly fewer hours outdoors than boys [19–22]. The longer life expectancy of females in most cultures could also contribute to a higher global burden of URE. The loss of accommodative ability and lens opacity with aging can lead to presbyopia and crystalline source refractive error among older people, which constitutes a large part of visual impairment due to URE. Moreover, the inequality of social, cultural, and economic status between men and women is believed to reduce access to eye care services, including refractive correction for women [23]. As estimated by one previous study, the median annual cost of refractive correction was 226.48 dollars (including eye exams and eyeglasses) in the United States, which could be a considerable sum for low-income adults [24]. However, the sex discrepancy of spectacle coverage has been reported as less extensive than expected [25, 26], which implies that the gender inequality of resource distribution may not be the primary cause.

The age-specific variation curve of DALY rates due to URE showed that the steepest increase in disease burden occurred in the 5–9 age group, followed by relatively smooth growth in the 10–14 age group, DALY rates then seemingly reached a plateau at 15–19 years of age. This trend was nicely consistent with the growth law of the ocular axis and myopia, which typically exhibits fast progression early at the age of 6–8 years, followed by slowing and eventual stabilization of refractive error in late adolescence [14, 15, 27]. The increasing study burden among these school-age children may explain the growing prevalence rate of visual impairment associated with URE [21, 28].

Regional inequality was also obvious, with a higher disease burden among adolescents in the WHO Eastern Mediterranean Region, Region of Americas and South-East Asia Region. Our results also revealed that regions with middle to high income and SDI have higher DALY rates. The HDI and SDI, two widely used indexes of socioeconomic development, were significantly positively correlated with visual impairment due to URE in univariate linear regression. The association of disease burden of URE with HDI and SDI might change with economic status. We compared the results of our disease burden analysis focused on adolescents to similar studies among individuals of all ages. Our results turned out to contrast with the findings of Lou et al., in which age-standardized DALY rates were inversely associated with HDI [10]. These differences could be attributed to the diverse spectrum of diseases at different ages. The aging process of ocular refractive structures, such as loss of lens power and lens opacity, can lead to presbyopia and crystalline source refractive error among older people, which constitutes a large part of visual impairment due to URE. By multiplying prevalence, the labor force participation rate, the employment rate, a disability weight and the GDP per capita, the potential productivity loss caused by 244 million uncorrected presbyopia cases among people aged < 50 years were estimated to reach US $11.023 billion (0.016% of global GDP) [29]. The prevalence of presbyopia is estimated to be higher in regions with longer life expectancies, whereas a greater burden of visual impairment resulting from uncorrected presbyopia occurs in less developed countries. The low amounts of available eye care resources and poor optical correction rates in these areas were proven to be the underlying causes [30]. Moreover, the quantity and quality of cataract surgery that could correct refractive errors to some extent were also positively associated with GDP per capita and HDI [31]. For adolescents under 20 years of age, a recent meta-analysis by Hashemi et al. estimated global and regional prevalence figures of refractive errors: the estimated pooled prevalence of astigmatism (> 0.50 D, 14.9%) was higher than that of myopia (≤ − 0.50 D, 11.7%) and hyperopia (≥ + 2.0 D, 4.6%). The prevalence and severity of astigmatism and hyperopia often decrease during emmetropization, while myopia tends to exhibit fast progression in school-age children [32]. This process can be greatly influenced by environmental risk factors. Low outdoor time, dim light exposure, close use of electronic screen (phones / laptops), education and living in an urban environment has been successively suggested as possible risk factors for myopia in adolescents in recent studies [13, 33]. Among these factors, education as an element of SDI and HDI plays an important role. The tendency for schooling to lead to an increased prevalence of myopia and visual impairment has been documented in almost all major population groups [21, 28, 34]. This result agrees with our finding that primary school dropout rates were inversely correlated with the DALY rates due to URE in both Pearson correlation and univariate linear regression to sunlight effectively prevents the development of myopia [11, 12]. Third, significant inequities in resources for refractive error correction still exist in developed countries with high urbanization rates [35, 36] and in urban areas of developing countries [37]. As a result, the need for refractive error correction in many people, especially migrants, remains unmet, although resources are adequate in these regions. This is partially attributable to socioeconomic barriers, such as low income, lower rates of health care coverage, fewer visits to health services, and language barriers [38, 39].analyses.

Additionally, variations in other parameters, such as living environment, diet and lifestyle accompanying societal development may also contribute to the high prevalence of URE and visual impairment among adolescents [13, 33]. Therefore, we explored the effects of primary school dropout rates and urbanization rates on URE burden with multiple linear regression analysis. A heavier disease burden due to URE in young people was suggested to occur among higher urbanized countries with lower primary school dropout rates. Potential mechanisms were enumerated as follows: first, primary and middle students could be more susceptible to myopia due to environmental factors such as heavy educational stress and fewer outdoor activities [21]. Second, findings from many studies indicated that the burden of myopia may be heavier among urban residents, which is consistent with our results [40–42]. Urban areas are usually characterized by more severe environmental pollution (less green space, ambient light exposure), different lifestyles (lower levels of time outdoors and higher levels of indoor activities) [41] and heavy academic pressure, all of which may affect refractive errors. Recent interventional prospective studies have shown that encouraging outdoor activity and exposure.

This study has several limitations. First, the data of URE burden in this study included myopia, hypermetropia, astigmatism and etc., thus, URE subtypes were not investigated. Second, HDI and SDI were used as indicators of socioeconomic condition, but these indices could not fully describe social resources. Third, the measurement of HDI and SDI are very similar and they may show strong correlation. Therefore, we applied stepwise linear multiple regression to eliminate collinearity. Fourth, besides HDI, SDI, education and urbanization, there are still numbers of risk factors which may relate to DALYs not included in this study due to lack of available data.

Despite the limitations mentioned above, the study findings can be useful for developing targeted strategies to address the severe visual impairment resulting from URE. The estimated large disease burden reveals a challenging task for healthcare policymakers, and considerable efforts will be required to scale up refractive error services for adolescents.

## Conclusions

In conclusion, the global visual impairment burden of URE among adolescents remained stable without significant alleviation from 1990 to 2019. Older age and female sex were associated with a higher burden of URE. The DALY rates rapidly increased in the 5–9 age group and reached a plateau at 15–19 years old adolescents. High socioeconomic development status (indicated by HDI and SDI), low primary school dropout rates and high urbanization rates were associated with increasing DALY rates. Based on the results, we recommend a universal screening program for 15–19 years old adolescents and an early warning system (EWS) for the 5–9 age group is also necessary for timely detection and control of refractive errors. Moreover, we should be aware of the growing disease burden of URE among adolescents along with the process of urbanization and universal education. These findings can provide information for policymakers to craft more targeted strategies to prevent, screen for and control adolescents’ visual impairment due to URE.

## Data Availability

The datasets generated and/or analysed during the current study are available in the Global Health Data Exchange (GHDx) repository,  http://ghdx.healthdata.org/gbd-results-tool and United Nations Development Programme repository, http://hdr.undp.org/. Global maps were generated from a data visualization tool available from the GHDx, https://vizhub.healthdata.org/gbd-compare/. Statistical analyses were performed using SPSS software (SPSS, Inc.; Chicago, IL, USA; version 25.0). Figures were drawn using GraphPad Prism software (version 7.0e, GraphPad Software; San Diego, CA, USA).

## Abbreviations

URE: uncorrected refractive error
DALYs: Disability-adjusted life years
YLDs: Years lived with disability
YLLs: Years of life lost
GBD: Global burden of disease
IHME: Institute for Health Metrics and Evaluation
WHO: World health organization
HDI: Human development index
SDI: Socio-Demographic Index
GNI: gross national income
ANOVA: one-way analysis of variance
